# Prevalence of Frailty and Its Standardised Assessment Tools among Malaysian Older Person: A Systematic Review

**DOI:** 10.21315/mjms2022.29.6.4

**Published:** 2022-12-22

**Authors:** Muhammad Iqbal Shaharudin, Nur Fatin Nabila Abd Rahman

**Affiliations:** 1 Physiotherapy Programme, Faculty of Health Sciences, Universiti Teknologi MARA, Pulau Pinang, Malaysia; 2 Academy of Language Studies, Universiti Teknologi MARA, Melaka, Malaysia

**Keywords:** frailty, older person, prevalence, standardised assessment tools, Malaysia

## Abstract

Frailty is conceptually defined as a state of increased vulnerability in older persons, resulting from age-associated declines in physiological reserve and function as they cope with everyday life stressors. To date, the prevalence of frailty has been assessed in various ways. The objective of this study was to identify the prevalence of the condition and the assessment tools used to determine its occurrence among Malaysian older individuals. A systematic literature search was performed on electronic bibliographic databases, namely, Web of Science, Scopus, EBSCOHost: MEDLINE Complete and Google Scholar. Ten eligible articles were reviewed and evaluated using the Preferred Reporting Items for Systematic Reviews and Meta-Analyses and the Newcastle-Ottawa Scale. Frailty was diagnosed using Fried’s frailty phenotype and the Groningen Frailty Indicator (GFI). Assessment tools that included physical performance tests detected a lower prevalence of frailty than that determined using questionnaire-based tools. The prevalence of frailty ranged from as low as 6% to as high as 76%, and there was a higher prevalence among older persons living in aged care homes. This review suggests increasing prospective and interventional studies on frailty to establish a cause–effect relationship between standardised tools of assessing frailty and its prevalence among Malaysian older persons and provide guidelines for health professionals on promoting active lifestyles among older populations.

## Introduction

Malaysia is expected to reach an ageing nation status by 2035, during which more than 15% of its population will have reached the age of 60 years old and older (1). Older persons are considerably susceptible to frailty because of their predisposition to adverse health effects (2, 3). Frailty is conceptually defined as a state of increased vulnerability in older individuals, resulting from age-associated declines in physiological reserve and function as they cope with everyday life stressors (4, 5). Regarded as the ‘holy grail’ of geriatric medicine (4), this syndrome has been associated with a high rate of comorbidity and a high risk of falls, hospitalisation, disability and mortality (3, 6–8). Frailty is dysregulation across multidimensional determinants, such as physical, cognitive, psychological and social determinants (4, 9). It is therefore an unavoidable part of the ageing process, which is not limited to older populations (9).

According to a systematic review, the global mean prevalence of frailty is 10.7% (10), which is close to the findings of a recent systematic review and meta-analysis which is 10% (11). Pooled studies of 62 countries and a systematic review conducted in China reported a prevalence of 12% (11, 12). Research also indicated variations in prevalence between genders, with rates being higher among females than their male counterparts (8, 11, 12). A global study reported that different types of assessment tools are used to identify the occurrence of frailty (11). These tools are the Fatigue, Resistance, Ambulation, Illnesses and Loss of Weight (FRAIL) scale, Fried’s criteria, Study of Osteoporotic Fractures (SOF) criteria, Comprehensive Frailty Assessment Instrument, Clinical Frailty Scale, Comprehensive Geriatric Assessment, Edmonton Frailty Scale, Groningen Frailty Indicator (GFI), the Identification of Seniors at Risk, Kaigo-yobo and Kihon checklists, Preferred Reporting Items for Systematic Reviews and Meta-Analyses (PRISMA), Strawbridge’s criteria, Tilburg Frailty Indicator and Vulnerable Elderly Survey-13 (11). A systematic review and meta-analysis in China reported the use of Fried’s frailty phenotype, the frailty index and the FRAIL scale in informing frailty (12).

As can be seen, a variety of methods are used to assess the prevalence of frailty among older people. To date, no study has pooled information regarding frailty studies in Malaysia. The objective of the current work was to identify the prevalence of frailty among the Malaysian older population and the tools used to assess the occurrence of the condition. The identification of such prevalence and corresponding assessment tools may provide evidence that can be used by the government to advance public health strategies.

## Methods

### Search Strategy

In December 2020, a systematic literature search was performed on Web of Science, Scopus, EBSCOHost: MEDLINE Complete and Google Scholar to identify prospective papers for review. The search was managed using the primary search terms (frail OR frailty) and the following secondary search terms: (older person OR older adult OR elderly OR senior OR geriatric OR aged OR ageing) AND (Malaysia OR Malaysian).

### Inclusion Criteria

Studies involving participants aged 60 years old and older and full-text articles written in English and published between January 2011 and April 2021 were included in the analysis. Studies that were conducted only in Malaysia, that were intended to inform the prevalence of frailty and that used validated frailty measurements that include physical performance tools were also deemed eligible for review. Finally, studies with participants who lived in either community dwellings or residential areas were covered in the analysis. The entries in the bibliographic databases were manually screened for duplication.

### Data Selection

Two reviewers (MIS and NFNAR) independently chose the articles for analysis on the basis of the inclusion criteria. The selection was conducted by screening article titles and abstracts, after which full texts were examined to determine their eligibility for inclusion. The initial sample of articles was compared in a consensus meeting and disagreement was resolved through discussion. Articles that both reviewers deemed suitable were included in the final sample. The selected articles were reviewed following PRISMA guidelines (13), and the study was registered in International Prospective Register of Systematic Reviews (PROSPERO; registration number: CRD42021228646) to avoid duplication and reduce reporting bias.

### Data Extraction

Data from the selected articles were retrieved using structured sheets containing demographic information, including ages, the total number of participants and the location of the studies. Information regarding standardised assessment and physical performance tools and the studies’ outcomes, limitations and strengths were also included.

### Study Evaluation

The reviewers used the Newcastle-Ottawa Scale to assess the methodological quality of the selected articles. Eight quality assessment tools with three main components (selection, comparability and exposure) were used to evaluate the cohort and case control studies. The maximum score that could be derived was 9 points and studies were considered high quality if they scored at least 5 points (14).

## Results

### Study Selection

**Figure 1:**
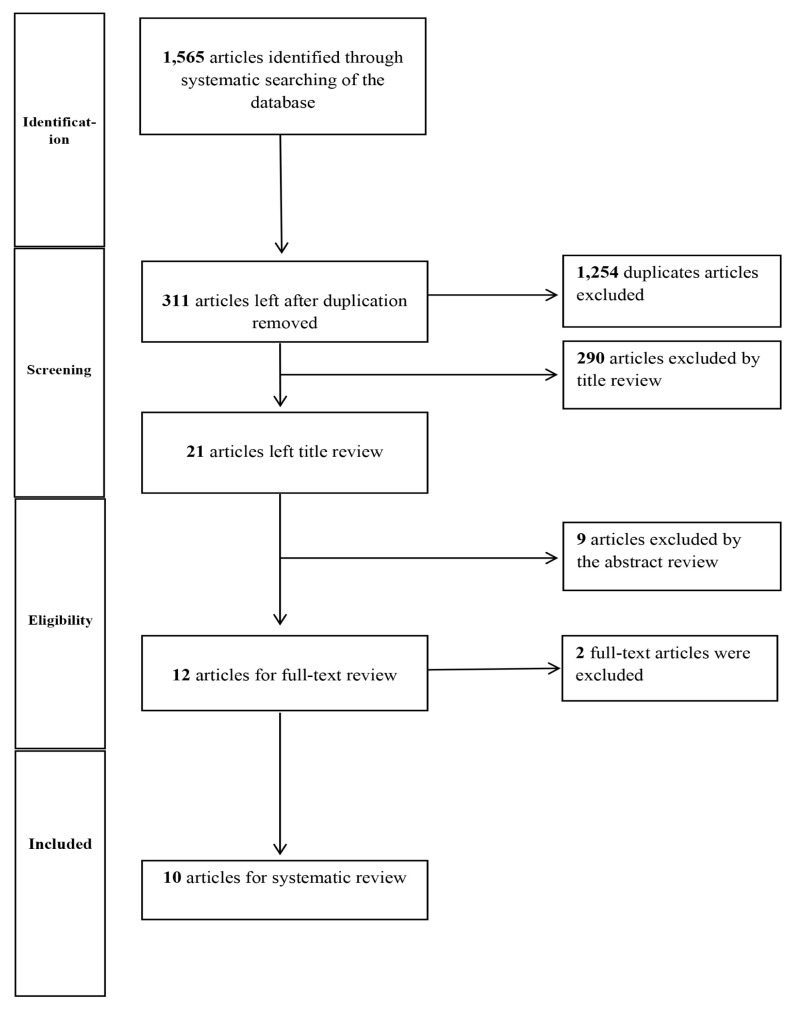
Study selection flowchart of published articles between the years 2011 and 2021

The database search yielded 1,565 articles, of which 1,254 were duplicate studies and therefore excluded. The titles and abstracts of the remaining 311 articles were screened, leaving 21 for potential inclusion. Following full-text assessment and screening on the basis of the inclusion criteria, 10 studies were deemed eligible for analysis. The PRISMA flowchart in Figure 1 illustrates the selection process.

### Study Characteristics

The characteristics of the 10 studies are summarised in Table 1. These studies were conducted in various states and regions of Peninsular Malaysia, namely Selangor, Negeri Sembilan, Terengganu, Perak and Kuala Lumpur. Eight studies were conducted in a single state (8, 15–21) and two were carried out in two states (20, 22). The specific contexts were an aged care home for three of the studies (20, 21, 23) and a residential area for the rest of the investigations. The majority of the participants lived in urban areas (15, 17–21). All the studies involved participants aged 60 years old and older, with most of them (15, 16, 18, 22) reporting a mean age of 65 years old and above; this age range approximates the definition of older persons by the Department of Statistics in Malaysia (24). Two studies involved participants with a mean age of 70 years old and older (16, 23).

### Prevalence of Frailty

The studies derived broadly varying results on the prevalence of frailty, with percentages ranging from as low as 6% (15) to as high as 76% (21). Three studies reported a prevalence of more than 45% (20, 21, 23). The prevalence of frailty was higher among participants living in residential settings (20, 21, 23) and the prevalence of falls was higher in studies that used non-physical performance tools in identifying the state of frailty (20, 21, 23). Seven studies indicated that females exhibited a higher prevalence of frailty than their male counterparts, with the mean value ranging from 50% to 70% (8, 11, 15–19, 22).

### Standardised Assessment Tools

In terms of tools for identifying frailty, the studies can be divided into those that adopted and excluded performance tests in their assessments. Seven studies determined frailty using either the modified or standardised version of Fried’s frailty phenotype (8, 15–19, 22), whereas the remaining three used the GFI (20, 21, 23). Fried’s frailty phenotype is a standard tool that entails conducting physical performance tests in assessments, and the GFI is a self-report screening instrument.

Standardised Fried’s criteria were used in three studies (17, 18, 22) and the modified version was used in four studies (8, 15, 16, 19). The modification of Fried’s criteria includes but is not limited to the incorporation of the Geriatric Depression Scale (GDS) for determining exhaustion phenotypes (8), multidimensional deficit accumulation model (FI) (15) and rapid assessment of physical activity for ascertaining physical activity levels (16). The application of cut-off points from the Cardiovascular Health Study (19) is also encompassed in the modified version of Fried’s frailty phenotype.

Other standardised assessment tools used in the study were cognitive assessments: the GDS, Mini-Mental State Examination, Montreal Cognitive Assessment, Digit Span, Rey Auditory Verbal Learning Test, Digit symbol forward and backward and Addenbrooke’s Cognitive Examination (19, 22). A study carried out physical senior fitness tests that involved the two-minute walk test, back scratch test, chair sit and reach test, the 30s sit to stand test, timed up and go (TUG) and single leg stance test (19). Other assessment tools used were Potentially Inappropriate Prescribing, Potentially Inappropriate Medication, Drug Burden Index, Pittsburgh Sleep Quality Index, KATZ Index of Independence in Activities of Daily Living (ADL) and Medication Appropriateness Index (MAI) (18, 21–23).

### Summary of Findings

Frailty was positively associated with advanced age (8, 16, 18, 22) and poor physical performance (15, 17, 19, 22), associated with existing medical conditions (8, 16–19) and significantly associated with cognitive ability (8, 15, 19, 22). Participants with a low socioeconomic status showed a high risk of susceptibility to frailty (8, 18). Two studies found that being female was positively related to frailty (8, 17), and a study on cognitive frailty indicated that advancing age, decreased TUG test performance and physical frailty were significant risk factors for the condition (22).

Frailty was also significantly associated with poor self-rated health, being single, low body mass index, abdominal obesity, physical disability, a history of falls, lack of balance, low peak respiratory rates, slow gait speeds, low skeletal muscle mass, high serum CRP levels and institutionalisation (8, 15–19). Studies that used GFI as a frailty assessment tool found that frailty was positively associated with very poor sleep quality, central nervous system (CNS) medication intake and a high Medication Appropriateness Index (MAI) score (23, 25).

## Discussion

As previously stated, this study was aimed at determining the prevalence of frailty and the standardised tools used to assess it among Malaysian older persons. This condition is widely prevalent in the country, and this prevalence is even higher among older individuals living in aged care homes—a situation similar to that described in a nationally representative study in the United States, where people in such institutions are twice as likely to be frail than those living in residential areas (26). A prospective study also indicated that living in a residential care setting is significantly associated with frailty, independently of comorbidity and functional limitation (27).

Variations in prevalence depend on the types of assessment tools used to identify frailty. This study uncovered that the use of self-report tools detected the prevalence of frailty to a higher degree than that achieved with physical performance tests. The findings are in line with a recent study that reported a higher prevalence when the condition is assessed using self-report questionnaires (28, 29). However, a few studies pointed to an underestimation of frailty when self-report questionnaires are used (30, 31). A recent meta-analysis study also found that a physical frailty test determines a lower percentage of frailty prevalence than that ascertained using a frailty index (11). There is also a higher prevalence of frailty among females than males, as supported in the past systematic review study. This result is attributed to lower lean body mass and muscle mass in females than in males (3, 10).

The advantage of self-report tools for determining frailty is their quick and easy implementation. Such tools do not require training and a unique instrument for administration. Even so, the validity of a self-report questionnaire is influenced by the cognitive level of a participant. Severe cognitive impairment may lead to inaccurate assessments of frailty (30). Conversely, physical performance tests enable access to the physical aspects of disability and clinicians can prescribe tailored interventions on the grounds of baseline characteristics (32). However, the drawbacks of physical screening are that it is time-consuming, reduces the feasibility of assessing a large population and requires special tools and training to administer.

The reviewed studies used two primary standardised assessments to identify frailty: Fried’s frailty phenotype and the GFI. Both include multiple components in identifying frailty, but the former involves physical performance testing, whereas the latter depends entirely on a self-report questionnaire. Nevertheless, the GFI features broader domains for identifying frailty, including physical, cognitive, social and psychological aspects.

Frailty is a multidimensional factor that is not limited to physical performance capabilities and components, including psychological, cognitive, clinical and social aspects (9, 33). A one-dimensional tool or self-report questionnaire may be helpful as an early method of identifying frailty but further screening that encompasses multidimensional factors is vital in deriving more accurate clinical findings and ensuring a holistic management of the condition.

## Conclusion

In summary, there are variations in the prevalence of frailty among afflicted individuals mainly because of the use of different assessment tools. Longitudinal and prospective studies may help to establish a cause-effect relationship with respect to frailty. Intervention studies on physical activity can also be conducted to provide guidelines for health professionals, especially physiotherapists, in implementing evidence-based practice as they promote the pursuit of active lifestyles among older persons. Frailty is reversible (9, 34) and engaging in an active lifestyle helps alleviate physically related health conditions in older individuals.

## Figures and Tables

**Table 1:** Prevalence of frailty and its standardised assessment tools among Malaysian older person

No	Article	Participant (age, *N*, location)	Prevalence of frailty	Standardised assessment tools (frailty)	Other standardised assessment tools	Result	Strength and limitation
1.	Ahmad et al. (8)	Age ≥ 60 years old *N* = 2,413 Location Kuala Pilah a district in the state of Negeri Sembilan, Malaysia	Overall 9.4% (95% CI: 7.8, 11.2) Men = 37.9% Women = 62.1%	Modified Fried’s frailty phenotype Weight loss (self-reported) Exhaustion (retrieved from Geriatric Depression Scale [GDS]) Low activity (assessed using Physical Activity Scale for Elderly [PASE]) Weakness (handgrip [Jammar dynamometer]) Slowness (4-metre walk test)	-	Frailty was significantly associated with older age, women and respondents with a higher number of chronic diseases, poor cognitive function and low socioeconomic status (*P* < 0.05)	Strength Large sample size and objective measurements of grip strength and walking speed Limitation Missing data
2.	Sathasivam et al. (15)	Age (mean ± SD) years old 69.6 ± 7.2 *N* = 789 Location An urban district in Malaysia	Overall 5.7% Men = 28.9% Women = 71.1%	Physical phenotype (Fried’s criteria) Multidimensional deficit accumulation model (FI)	-	Physical disability falls, and cognition is important determinants for frailty	Strength Identifies potential risk factors for frailty and provide early preventive measures for prefrail state individual Limitation Multistage cross-sectional study: causal relationship could not be established
3.	Mohd Hamidin et al. (16)	Aged (mean ± SD) years old 73.32 ± 6.05 *N* = 279 Location Districts of Terengganu, Malaysia.	Overall 18.3% Men = 41.2% Women = 58.8%	Modified Fried’s frailty phenotype. Unintentional loss of weight Feeling of exhaustion from Centre for Epidemiologic Studies Depression (CES-D) scale Weakness was defined as mean grip strength Slowness was defined as usual walking speed Low physical activity level assessed by Rapid Assessment of Physical Activity (RAPA) questionnaires	-	The frail older adults were positively associated with advanced age, being unmarried, hospitalisation in the previous year, poor self-rated health, and lower body mass index	Strength Baseline data and deepen the knowledge of frailty Limitation A cross-sectional study: causal relationship could not be established A small number of participants limit the generalisability
4	Badrasawi et al. (17)	Age ≥ 60 years old Men 68.9 ± 5.9 Women 67.3 ± 5.7 *N* = 473 Location Klang Valley of Malaysia	Overall 8.9% Men = 26.2% Women = 73.8%	Fried’s criteria Unintentional weight loss of 5 kg and above over the last year Weakness (handgrip) Exhaustion and poor endurance and energy from the CES-D scale Slowness (gait speed) Low physical activity of the PASE	-	Binary logistic regression analyses showed that female gender, abdominal obesity, low peak respiratory flow rate score and slower rapid pace gait speed were significant predictors of frailty	Strength The study has highlighted the prevalence and risk factors of frailty from a wide range of determinants Limitation A cross-sectional study: causal relationship could not be established The study did not explore this relationship as it. focused on frailty as an outcome and not as a disability
5.	Norazman et al. (18)	Age ≥ 60 years old *N* = 301 Location Older person residing at People Housing Project (Projek Perumahan Rakyat (PPR) at Kuala Lumpur	Overall 15.9% Men = 22.9% Women = 77.1%	The standardised phenotype of frailty proposed by Fried	-	Frailty can be predicted by increasing age, low household. income, being at risk of malnutrition, as well as having a low skeletal muscle mass and high serum CRP level	Strength Identifies prevalence of frailty among urban community elderly with low socioeconomic background and the multiple components of the frailty syndrome Limitation The prevalence values reported in these studies are the representative sample of the older adult population The nature of an observational study is limited to the interpretation and the cause-effect mechanism of the current findings
6.	Murukesu et al. (19)	Aged ≥ 60 years old *N* = 302 Location Older person residing at Projek Perumahan Rakyat (PPR) at Kuala Lumpur	Overall 40.7% Men = 31.1% Women = 68%	Fried’s criteria using cut of points as outlined in the cardiovascular health study	GDS score The Mini-Mental State Examination (MMSE) Addenbrooke’s Cognitive Examination (ACE-III). Functional fitness status was determined using the senior fitness test	Frailty is highly prevalent among Malaysian institutionalised older adults. Hypertension, cognitive impairment and lower dynamic balance and mobility were found to be risk factors of frailty	Strength The study provides insight into the prevalence of frailty, its associated factors, and the cognitive and functional status among the ethnically diverse Malaysian older adults residing in institutions Limitation The causal relationship between the significant factors associated with frailty could not be formed due to the cross-sectional study design
7.	Rivan et al. (22)	Age (mean ± SD) years old 67.00 ± 4.98 *N* = 282 Location Selangor and Perak (representing central and northern regions of Malaysia)	Overall 35.5% Men = 37% Women = 63%	Standardised phenotype of frailty proposed by Fried	PASE Cognitive assessments (MMSE, MoCA, Digit span, RAVLT, Digit symbol, VR I, and VR II)	Advancing age and depression have a significant role in the development of CF. Ageing is an established predominant risk factor for both frailty and cognitive impairment A one-unit increase in the TUG test significantly increases the odds of developing CF among an older population Physical frailty predicts CF better than MCI does	Strength Report on the incidence rate of cognitive frailty among older adults in Malaysia using longitudinal data The study involved a wide range of parameters with a detailed protocol covering several domains: fitness, cognitive function, nutrient intake, anthropometric measurements, body composition, psychosocial function, and biochemical indices as predictors of cognitive frailty Limitation The study only involved. Two out of 14 states in Malaysia, with a smaller sample size at the follow-up, do not match the Malaysian population’s national composition
8.	Hasan et al. (20)	Aged ≥ 65 years old *N* = 202 Location A total of 17 private aged care homes around Klang Valley in Malaysia	Overall 76%	Groningen Frailty Indicator (GFI)	Potentially Inappropriate Prescribing (PIP) Potentially Inappropriate Medication (PIM)	The number of medications used per participant correlated significantly and positively (0.21, P = 0.002) with a GFI score	Strength Identification of medication appropriateness and frailty among residents of aged care homes in Malaysia Limitation Cross-sectional the design does not allow the establishment of any cause-effect relationship The small number of participants from aged care homes in urban central Peninsular Malaysia
9.	Kumar et al. (23)	Aged mean ± SD (74.5 ± 8.4) years old *N* = 151 Location Residents of 11 aged care homes in three states in Malaysia	Overall 75.5%	GFI	Pittsburgh Sleep Quality Index (PSQI) Drug burden index (DBI) PIM PIP	The study population with very poor sleep quality (VPSQ) had the highest mean GFI score (4.9 ± 2.5), followed by participants with moderately poor sleep quality (MPSQ) (4.5 ± 2.8) and participants with normal sleep quality (NSQ) (2.3 ± 2.4).	Strength The study establishes a relationship between sleep quality and frailty, a physical health outcome Limitation It is a convenience sampling that may lead to selection bias The study has a small number of non-Chinese participants limits generalisability to the (whole) Malaysian population
10.	Hasan et al. (20)	Aged ≥ 65 years old *N* = 202 Location A total of 17 private aged care homes around Klang Valley in Malaysia	Overall 46.9%	GFI	KATZ ADL index, Medication Appropriateness Index (MAI) and PIM	There is a high prevalence of frailty in residents taking CNS medications The level of medication appropriateness assessed using the MAI was higher among frail participants	Strength This study confirms the GFI’s construct validity and internal consistency for measuring frailty in elderly residents of aged care homes Limitation Unable able to establish causal relationships between frailty and medication appropriateness
